# Beyond the Bone Health: A Narrative Review Unveiling the Role of Bisphosphonates in Reducing the Risk of Myocardial Infarction

**DOI:** 10.7759/cureus.80089

**Published:** 2025-03-05

**Authors:** Pakeezah Tabasum, Muhammad Umar, Riya Mary Richard, Saba Khan, FNU Momna, Durr e Shahwar, Ayesha Hidayat, Abdulqadir J Nashwan, Waseem Sajjad, Waleed Inayat Mohamed

**Affiliations:** 1 Department of Medicine, Peoples University of Medical and Health Sciences for Women, Nawabshah, PAK; 2 Department of Medicine, Khairpur Medical College, Khairpur, PAK; 3 Faculty of Medicine, Ivane Javakhishvili Tbilisi State University, Tbilisi, GEO; 4 Department of Nursing and Midwifery Research, Hamad Medical Corporation, Doha, QAT; 5 Department of Medicine, King Edward Medical University, Lahore, PAK; 6 Department of Internal Medicine, Holy Family Hospital, Rawalpindi, PAK

**Keywords:** alendronate, anti-atherosclerosis, bisphosphonates, myocardial infarction, vascular calcification

## Abstract

Bisphosphonates, a class of anti-osteoporotic drugs, are known to inhibit bone resorption and reduce fracture risk. This review evaluates the evidence for their use and also aims to assess their potential benefits, safety, and effectiveness in reducing the risk of myocardial infarction. Bisphosphonates, in addition to their conventional role in bone health, reduce the accumulation of calcium within vascular walls. Therefore, they are known to decrease the potential risk of myocardial infarction in patients with a higher likelihood of cardiovascular problems. However, they may exacerbate other cardiac issues and do not appear to reduce mortality rates. Bisphosphonates, when used as an anti-osteoporotic drug, generally have a favorable safety profile in patients with osteoporosis, regardless of treatment duration, age, sex, or underlying cardiovascular conditions. Nonetheless, strict adherence to bisphosphonate therapy over a longer duration may help reduce the risk of myocardial infarction.

## Introduction and background

Bisphosphonates (BPs) are the most widely prescribed anti-osteoporotic drugs. This review examines the effects of BPs on vascular disorders and their possible mechanisms of action. While overall data remains ambiguous, some studies suggest that BPs may offer cardiac protective advantages in the case of myocardial infarction (MI). Our study explores the potential anti-atherosclerotic properties of BPs, particularly their role in reducing vascular calcification of coronary arteries and other possible mechanisms that could reduce the risk of MI [1]. BPs have long been recognized for their effectiveness in treating osteoporosis by blocking bone resorption and increasing bone mineral density, which helps in reducing osteoporotic fractures [2]. Calcification is crucial to bone health; low bone mineral density is linked to a higher risk of cardiovascular events and an elevated risk of cardiovascular death [3]. A cohort study conducted in 2015 found that patients with osteoporosis are more prone to developing coronary heart disease compared to those without osteoporosis. Specifically, osteoporosis patients had a higher incidence of cardiovascular problems, with 23.5 per 1,000 person-years compared to 16.7 per 1,000 person-years in the comparison cohort [4].

The safety and efficacy of BPs in patients with MI have been subjects of recent debate and investigation. MI presents unique challenges in managing osteoporosis due to concerns about cardiovascular safety and potential drug interactions with MI treatments. Recent studies, including a meta-analysis by Kim et al., have explored the association between BP use and cardiovascular events, highlighting potential risks in MI patients [5]. Research by Abrahamsen et al. and Vestergaard et al. have also investigated the relationship between BP therapy and atrial fibrillation, suggesting a possible link that requires further examination [6,7]. Additionally, a cohort study by Park and Ko emphasizes the importance of considering comorbidities when assessing the risk of atrial fibrillation associated with BP use, underscoring the need for comprehensive patient evaluation [8].

This narrative review aims to gather available evidence on the safety and efficacy of BPs in patients at risk for MI. By reviewing research, observational studies, meta-analyses, and case reports, we seek to understand the potential benefits and risks of BP treatment in this patient population. However, literature defining the safety mechanisms of BPs in the context of MI and their incidence is limited. We aim to identify knowledge gaps by examining recent studies on cardiovascular safety related to MI, the cardiovascular adverse events associated with various BPs, and mortality rates. Our goal is to propose future research opportunities to enhance clinical decision-making and patient care. BPs appear to reduce the risk of MI regardless of treatment duration, age, sex, and baseline cardiovascular problems, with a lower incidence of MI observed when administered over a longer duration.

## Review

Mechanism of action and immunomodulatory effects

The mechanisms by which BPs exert their effects vary between their two categories based on the presence or absence of a nitrogen atom in their side chains. Nitrogen-containing bisphosphonates (NBPs) such as alendronate and zoledronate and non-nitrogen-containing bisphosphonates (NNBPs) such as clodronate and etidronate have distinct mechanisms of action [9]. NBPs, in particular, achieve their therapeutic effects by binding to calcium-rich regions within the skeleton. They accumulate in bone tissue due to their strong affinity for bone hydroxyapatite and their non-hydrolyzable P-C-P structure [10,11]. This accumulation leads to their uptake by osteoclasts during bone resorption, resulting in cytotoxic effects and a prolonged anti-resorptive impact [11]. NNBPs or first-generation BPs exert their effects by attaching to osteoclasts once they enter the bone, subsequently inhibiting ATP-dependent enzymes and inducing osteoclast apoptosis [12]. BPs act as stable analogs of pyrophosphate, an endogenous regulator of calcium metabolism [13].

In contrast, NBPs inhibit farnesyl pyrophosphate synthase (FPPS), a key enzyme in the mevalonate pathway. This inhibition reduces the synthesis of isoprenoid lipids and protein prenylation, affecting angiogenesis, endothelial cell survival, proliferation, migration, and tube formation [14]. Clinically, BPs significantly impact bone metabolism by inhibiting turnover, improving remodeling, and minimizing resorption. This reduces the risk of pathological fractures and increases bone mineral density, especially in osteoporosis patients [15]. Additionally, BPs are used to treat various skeletal conditions such as Paget's disease, multiple myeloma, and osteogenesis imperfecta, demonstrating a substantial reduction in the risk of bone fractures, particularly in postmenopausal women [16,17].

Beyond their traditional role in bone health, BPs exhibit multifaceted effects, including immunomodulatory actions that influence cytokine profiles. They inhibit nuclear factor kappa B (NFκ-B), reduce metalloproteinase activity, and suppress adhesion molecule expression [12,18]. These actions encompass anti-inflammatory properties and significant anti-arthritic effects, notably through the stimulation of synovial macrophage apoptosis [17,18]. For example, clodronate, a type of BP, has shown promise in managing arthritis by suppressing proteoglycan joint loss and regulating inflammatory mediators [19]. Moreover, BPs extend their inhibitory effects to vascular calcification as they accumulate in healthy, damaged, or diseased cells, reducing calcium deposits in the vascular wall and mitigating atherosclerotic progression through various mechanisms [20-22]. These findings highlight the remarkable versatility of BPs as therapeutic agents beyond their traditional role in bone health [23], as summarized in Table 1. Overall, the protective effects of BPs lie in their anti-atherosclerosis property via anti-inflammatory effects and reduced vascular calcification, which reduces the risk of MI. On the other hand, calcium deregulation, potentially leading to arrhythmias in high-risk populations such as those with previous cardiovascular disease, may contribute to adverse cardiac outcomes [8,21].

**Table 1:** Mechanism of action of bisphosphonates

	Nitrogen-containing bisphosphonates	Non-nitrogen-containing bisphosphonates
Site of attachment	Calcium-rich region with the bone [10]	Osteoclast cell within the bone [12]
Mechanism of action	Inhibit farnesyl pyrophosphate synthase, a key enzyme in the mevalonate pathway [14]	Inhibit ATP-dependent enzymes [12]
Biological effect	Reduce the production of isoprenoid lipids and protein prenylation [14]	Osteoclast apoptosis [12]​​​​​​​
Outcome	Inhibit angiogenesis, endothelial cell survival, proliferation, migration, and tube formation [14]	Inhibit bone resorption [14]

Anti-atherosclerotic properties of BPs

**Figure 1:**
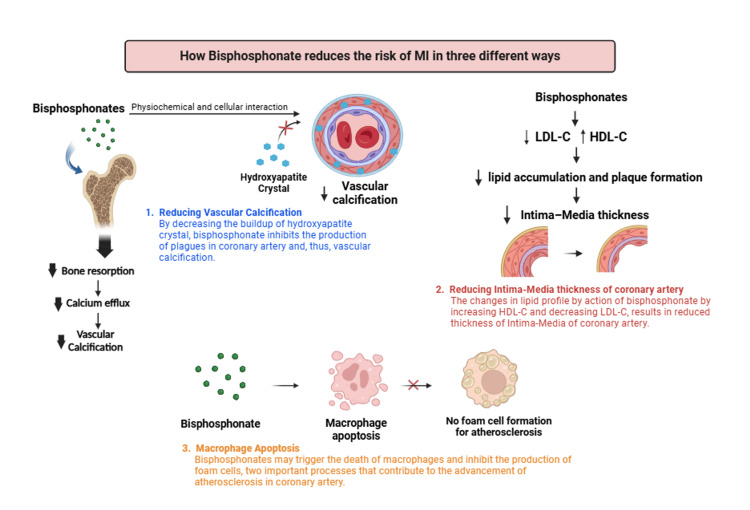
Mechanism of bisphosphonates in reducing the risk of myocardial infarction through an anti-atherosclerosis property Image Credits: Pakeezah Tabasum

BPs inhibit FPPS, a key enzyme in the mevalonate pathway that links bone and lipid metabolism. This inhibition helps slow the advancement of ectopic calcification. Additionally, BPs affect atheroma production, slowing down plaque formation in arteries [24]. It is hypothesized that there is an overlap in the pathophysiological mechanisms underlying atherosclerosis and osteoporosis, which can be summarized in three main mechanisms (see Figure 1). Firstly, BPs stimulate osteoblast differentiation and reduce bone resorption, decreasing calcium efflux from bones. This reduction in calcium efflux helps decrease vascular calcification [25]. Secondly, BPs enhance osteoblast differentiation, increasing osteoprotegerin production (OPG). OPG protects against vascular calcification, and its depletion can accelerate atherosclerotic lesion progression [26,27]. A study by Caffarelli et al. suggested that BPs reduce lipid accumulation by decreasing low-density lipoprotein (LDL) levels and increasing high-density lipoprotein (HDL) levels, which can reduce the thickness of the intima-media of coronary arteries [28].

Furthermore, research by Santos et al. demonstrated that one year of treatment with alendronate and etidronate significantly reduced carotid artery intima-media thickness, indicating an inhibitory effect on atherosclerosis [29]. BPs also target macrophages and monocytes, as shown in both in vitro and in vivo studies. They induce apoptosis in these cells and inhibit foam cell formation: two critical steps in the development of atherosclerosis [30].

Clinical safety vs. adverse effects of BPs

Evaluating the cardiovascular safety profile of BPs is crucial. Research has documented various cardiovascular outcomes among BP users, including atrial fibrillation, angina pectoris, coronary artery disease, and cardiac arrhythmias. The cardiovascular safety, adverse effect, and mortality rate of different BP drugs have been demonstrated below. Despite the exacerbation of some cardiac side effects, BPs are associated with a potential reduction in the risk of MI [31,32]. The findings from studies on MI and BPs vary widely. Generally, the risk of MI appears reduced with strict adherence to BP therapy, particularly in the absence of underlying cardiovascular comorbidities. Observational studies provide nuanced insights into the role of BPs as anti-osteoporosis therapy in patients with cardiovascular conditions. For instance, a Danish cohort study reported a 33% reduction in cardiovascular event risk among patients receiving oral BPs compared to controls [33]. Conversely, a Swedish and Danish study using propensity score matching revealed increased risks of heart failure and arrhythmia with zoledronic acid use. However, there was no significant increase in cardiovascular mortality [34].

Disentangling causality remains complex due to inherent baseline risks that obscure clear interpretations. Investigations into major adverse cardiovascular events (MACE) present a mixed picture, showing an overall neutral stance but with a heightened risk of atrial fibrillation [22]. The World Health Organization's pharmacovigilance study highlighted adverse effects such as arrhythmias while paradoxically suggesting a potential mitigating effect on MI [31]. Cohort studies focusing on atherosclerotic cardiovascular events offer promising observations, indicating reduced cardiovascular and coronary events incidents with consistent BP use [35]. However, nested case-control studies suggest that while BP use may not significantly increase cardiovascular risk in older individuals, closer monitoring is needed for patients with pre-existing cardiovascular or cerebrovascular diseases [36]. Studies by Billington and Reid recommend early intervention with BPs for greater effectiveness, supported by rigorous randomized controlled trials and meta-analyses [15]. Specific patient populations, such as breast cancer survivors, also warrant nuanced considerations [37]. Despite some studies suggesting potential cardioprotective effects, the overall evidence supporting BP use in cardiovascular disease remains inconclusive. Meta-analyses have cast doubt on the purported benefits, implying that BPs should primarily be recommended for bone-related conditions like osteoporosis and fractures [38]. Moreover, while one meta-analysis indicates no significantly increased risk of MI with zoledronic acid treatment, it highlights an elevated risk for atrial fibrillation and arrhythmias [32]. Epidemiological investigations underscore the complex relationship between BP use and cardiovascular outcomes. While some studies suggest potential benefits, such as reduced all-cause mortality in specific subgroups, others do not support these findings. In conclusion, while BPs may offer potential anti-atherosclerotic benefits, carefully considering individual risk profiles is essential due to cardiovascular safety's complexities.

Table 2 lists the safety profile, adverse effect, and mortality rate of BP drugs.

**Table 2:** Safety profile, adverse effect, and mortality rate of bisphosphonate drugs SR: strontium ranelate; SERM: selective estrogen receptor modulator; MI: myocardial infarction; MACE: major adverse cardiovascular events

Drug	Cardiovascular safety	Cardiovascular adverse event	Mortality rate
Alendronate	It is regarded as the first-line choice for osteoporosis treatment. When comparing the risk of MI, other bisphosphonates, SR and SERM, found a 10% increased risk of MI compared to alendronate [39]	Increases the risk of atrial fibrillation in women with alendronate [40]	34% lower mortality rate was experienced as compared to non-users [38]
Zoledronate	It indicates a significantly reduced rate of cardiovascular events when compared with placebo, i.e., 2.9% of patients in the zoledronic acid group had incidents of MI than 11.1% of placebo [41]	An elevated risk of atrial fibrillation and arrhythmia was found, while no increased risk of MACE, angina, and heart failure [32]	15% lower mortality rate was declared when compared with the control group [42]
Risedronate	When comparing the weekly 35 mg and daily 5 mg risedronate dosing regimens, it was found that the weekly regimen was non-inferior in efficacy and tolerability to the daily dosing regimen [43]	The rate of cardiovascular events, coronary artery disease, and stroke had no difference when compared with placebo [44]	It is associated with a 10% reduction in the overall mortality rate [38]
Ibandronate	It has unrecognized risk for significant heart problem; however, it can reduce the sensitivity to detect pro-arrhythmia for drugs, when administered to older population [45]	Ibandronate is shown to have increased risk of ventricular ectopy and arrhythmia [45]	No study specifically discussed mortality rate, but it was found that bisphosphonate users with prevalent vertebral deformity had increased mortality rate [46]

Comparison of BPs with other anti-osteoporotic drugs

Calcium supplements are commonly used in osteoporosis patients; however, excessive intake can lead to calcification of arteries and soft tissues, formation of atherosclerotic plaques, and increased cardiovascular morbidity [47]. Bolland et al. found an increased risk of MI in patients taking calcium supplements compared to those receiving a placebo [48]. Saag et al. conducted a randomized controlled trial comparing the anti-osteoporotic drug romosozumab with alendronate (a BP). They reported that 2.5% of patients in the romosozumab group experienced serious cardiovascular events, including 0.8% with cardiac ischemic events. In contrast, 1.9% of patients in the alendronate group experienced serious cardiovascular risks, with 0.3% having cardiac ischemic events, and the findings were statistically significant in the study [49]. A systematic review and meta-analysis analyzed the cardiovascular events associated with BPs compared to denosumab, another anti-osteoporotic drug, finding that the denosumab group experienced more cardiovascular events [50]. Conversely, Choi et al. evaluated the safety of BPs for cardiovascular disease compared to denosumab, finding similar safety profiles [51]. Additionally, a retrospective cohort study by Wu et al. found improved overall survival in osteoporosis patients taking BPs compared to those not on BP therapy [21].

Consideration of confounding factors with BPs

The relationship between BPs and cardiovascular disease reflects a dual aspect: potential atherosclerotic protection and a possible increased risk of atrial fibrillation [23]. Atherosclerosis, a leading cause of cardiovascular illness in old age, involves the rupture of atherosclerotic plaques and subsequent arterial thrombosis, which can result in conditions such as heart attack or stroke [36]. Rodríguez et al.'s study found a 33% reduction in cardiovascular mortality risk with oral BPs compared to placebo [33]. NNBPs have been associated with a higher incidence of arterial calcifications in older women suggesting an age-dependent relationship between these medications and atherosclerosis [23]. Besides, the efficiency of BPs is reduced in patients with pre-existing cardiovascular issues [36]. Other than this, cardiac arrhythmias are more commonly reported with intravenous BPs, likely due to their impact on proteins regulating sarcoplasmic reticulum calcium levels, which may contribute to atrial fibrillation [22,31]. Specifically, intravenous zoledronic acid has been linked to an increased risk of heart failure compared to oral BPs [33]. Overall, BPs can lower the risk of MI and can be safely prescribed to osteoporosis patients. However, considerations regarding age, duration of treatment, and underlying comorbidities are essential to mitigate potential cardiovascular risks.

Limitations

The limitations of our review lie in our studies with different follow-up durations and differences in outcome. Additionally, our studies do not account for past lifestyle factors such as smoking, alcohol consumption, health-seeking behavior, and the use of antihypertensive or anticoagulant medications, all of which could influence cardiovascular events. Furthermore, while MI was reported in some studies, it was not consistently considered as a specific outcome across all included research, which focused broadly on cardiovascular events.

Recommendations

BPs can reduce the risk of MI when treatment duration, age, sex, and underlying cardiovascular conditions are considered. Strict adherence to BPs is recommended to mitigate cardiac adverse events, though careful monitoring is essential for patients with pre-existing cardiovascular issues. Special attention is required for elderly patients on BPs due to their increased risk of adverse effects [36]. Findings suggest that longer use of BPs is associated with a reduced likelihood of hospitalization for cardiovascular disorders. Adherence to BP therapy should be monitored, with the proportion of days covered (PDC) being a relevant metric [35]. After initiating treatment, bone mineral density should be assessed every two years or more frequently for high-risk individuals. For patients with a history of Paget's disease, alkaline phosphatase levels should be monitored 6-12 weeks after treatment begins and then every 6-12 months [52]. Given the biases in existing studies and the lack of robust clinical trials focusing on high-risk cardiovascular patients, caution is advised when prescribing BPs, especially to those with atrial fibrillation and arrhythmias. Alternative treatments such as denosumab, selective estrogen receptor modulators (SERMs), parathyroid hormone analogs (e.g., teriparatide), and calcitonin may be preferable for managing osteoporosis and other bone conditions in these populations.

Future directions

Despite the promising mechanisms suggesting that BPs may lower the risk of MI, several concerns remain unresolved. Further investigation is needed to determine whether BPs can effectively reduce the risk of other cardiovascular events, such as heart failure and stroke, both with and without underlying conditions. More randomized controlled trials are necessary to validate the efficacy of BPs in cardiovascular events. Additionally, it is crucial to ensure that research studies adequately support patient follow-ups. This includes tracking cardiovascular event rates among patients adhering to their medication regimens as well as those who take drug holidays.

## Conclusions

This study found that the beneficial effects of BPs on cardiovascular risk are diminished. The data regarding cardiovascular outcomes is conflicting, revealing that BPs may increase the risk of heart failure, arrhythmias, and other cardiovascular events. However, they seem to lower the risk of MI by slowing atherosclerosis progression. The review examines the impact of BPs on cardiovascular events and their possible mechanisms. While the overall data remains ambiguous, some studies suggest that BPs may offer cardiac protective benefits. The review highlights the importance of considering patient comorbidities in the evaluation and suggests that alternative treatments might be preferable for managing bone conditions in high-risk populations.
